# The Anti-Tumor Activity of the NEDD8 Inhibitor Pevonedistat in Neuroblastoma

**DOI:** 10.3390/ijms22126565

**Published:** 2021-06-18

**Authors:** Jennifer H. Foster, Eveline Barbieri, Linna Zhang, Kathleen A. Scorsone, Myrthala Moreno-Smith, Peter Zage, Terzah M. Horton

**Affiliations:** 1 Texas Children’s Cancer and Hematology Centers, Department of Pediatrics, Section of Hematology and Oncology, Baylor College of Medicine, Houston, TX 77030, USA; exbarbie@txch.org (E.B.); lxzhang@texaschildrens.org (L.Z.); scorsone@bcm.edu (K.A.S.); mxmoren3@texaschildrens.org (M.M.-S.); 2 Department of Pediatrics, Division of Hematology-Oncology, University of California San Diego, La Jolla, CA 92024, USA; pzage@ucsd.edu; 3 Peckham Center for Cancer and Blood Disorders, Rady Children’s Hospital, San Diego, CA 92123, USA

**Keywords:** pevonedistat, cell cycle, xenograft, rereplication, cullin–ring ligase, ubiquitination

## Abstract

Pevonedistat is a neddylation inhibitor that blocks proteasomal degradation of cullin–RING ligase (CRL) proteins involved in the degradation of short-lived regulatory proteins, including those involved with cell-cycle regulation. We determined the sensitivity and mechanism of action of pevonedistat cytotoxicity in neuroblastoma. Pevonedistat cytotoxicity was assessed using cell viability assays and apoptosis. We examined mechanisms of action using flow cytometry, bromodeoxyuridine (BrDU) and immunoblots. Orthotopic mouse xenografts of human neuroblastoma were generated to assess in vivo anti-tumor activity. Neuroblastoma cell lines were very sensitive to pevonedistat (IC50 136–400 nM). The mechanism of pevonedistat cytotoxicity depended on p53 status. Neuroblastoma cells with mutant (p53^MUT^) or reduced levels of wild-type p53 (p53si-p53) underwent G2-M cell-cycle arrest with rereplication, whereas p53 wild-type (p53^WT^) cell lines underwent G0-G1 cell-cycle arrest and apoptosis. In orthotopic neuroblastoma models, pevonedistat decreased tumor weight independent of p53 status. Control mice had an average tumor weight of 1.6 mg + 0.8 mg versus 0.5 mg + 0.4 mg (*p* < 0.05) in mice treated with pevonedistat. The mechanism of action of pevonedistat in neuroblastoma cell lines in vitro appears p53 dependent. However, in vivo studies using mouse neuroblastoma orthotopic models showed a significant decrease in tumor weight following pevonedistat treatment independent of the p53 status. Novel chemotherapy agents, such as the NEDD8-activating enzyme (NAE) inhibitor pevonedistat, deserve further study in the treatment of neuroblastoma.

## 1. Introduction

Neddylation is an ATP-dependent process which is involved in the regulation of intracellular protein destruction. Similar to the process of ubiquitination, neddylation is controlled by a multistep process involving three enzymes: a single activation enzyme (E1) known as the NEDD8-activating enzyme (NAE), several NEDD conjugating enzymes (E2) which bring NEDD8 to the targeted substrate protein, and many NEDD8/ubiquitin ligases (E3) which transfer and ligate NEDD8 to the targeted substrate. Recent studies have shown that inhibition of the E1-NAE disrupts cancer cell proliferation by interfering with cullin–RING ligase (CRL) mediated neddylation and subsequent ubiquitination [1,2,3,4]. Cullin–RING ligases (E3), the largest complex of ring ligases, are responsible for the timed degradation of many proteins involved in the cell cycle, including WEE1, Crlf2, Ets2 and I-κB. CRLs play an essential role in targeting proteins for ubiquitin-mediated destruction. Neddylation comprises nearly 20 percent of ubiquinated proteins. In addition to providing scaffolding for the ubiquitination of cell cycle proteins, CRLs also affect the degradation of proteins involved in hypoxia (HIF1α), signal transduction (p-53, I-κB), and DNA replication [5].

The neddylation inhibitor pevonedistat (MLN4924, TAK-924) inhibits the degradation of key regulatory proteins by blocking the ubiquitin-mediated proteasomal degradation pathway. Unlike proteasome inhibitors, pevonedistat blocks the degradation of only a subset of proteins degraded by the proteasome, the undergoing neddylation and subsequent degradation through cullin–ring ligase (CRL) ubiquitin substrates [1,2,3,4]. Pevonedistat has completed initial clinical testing in adults with solid tumors (NCT00677170), myeloma and lymphomas (NCT00722488), and hematologic malignancies (NCT00911066), and there has been interest in determining if neddylation inhibition can reduce tumor growth in pediatric tumors (NCT03323034).

Neddylation inhibition with pevonedistat is effective against many adult carcinomas both in vitro and in early clinical trials. Pevonedistat was initially noted to effectively kill colon carcinoma cell lines (4.3 μM) [1]. Pevonedistat was also shown to effectively inhibit DNA replication in a variety of adult and pediatric tumors (melanoma, ovarian tumors, multiple myeloma, diffuse large B cell lymphoma, and acute myeloid leukemia) [1,3,4,6,7,8]. The Pediatric Preclinical Testing Program (PPTP) showed potent activity when pevonedistat was tested against both an in vitro cell line panel as well as solid tumor xenografts, with increased event free survival (EFS) against Wilms tumor, glioblastoma, rhabdomyosarcoma, and neuroblastoma [9]. Here we evaluated the effect and mechanism of action of pevonedistat in neuroblastoma cell lines and orthotopic neuroblastoma mouse models

## 2. Results

### 2.1. Pevonedistat Is a Potent Inhibitor of Neuroblastoma Cell Viability In Vitro

**Figure 1:**
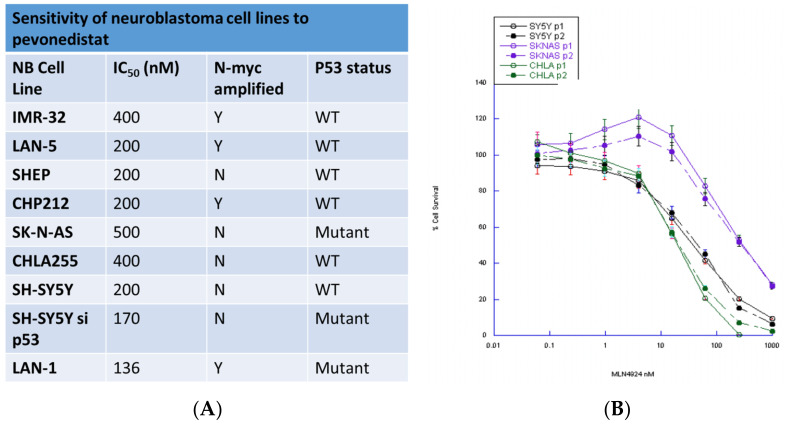
Sensitivity of neuroblastoma cell lines to pevonedistat. (**A**) Summary of neuroblastoma cell lines. (**B**) Dose-response curves for representative cell lines.

Using a panel of neuroblastoma cell lines that differ in *MYCN* and p53 status, we showed that pevonedistat was cytotoxic in vitro at nanomolar concentrations (Figure 1). All neuroblastoma cell lines tested were sensitive to pevonedistat with IC_50_ values ranging from 136–400 nM. Both p53 mutant (p53^MUT^) and p53 wild type (p53^WT^) neuroblastoma cell lines were sensitive to pevonedistat, and sensitivity did not vary by *MYCN* status (Figure 1).

We also assessed whether pevonedistat-induced cytotoxicity in neuroblastoma cell lines was dependent on p53 status. We created two p53 knockdown cell lines that could be compared to the parent cell with wild-type p53 activity. We tested the activity of pevonedistat in SY5Y (p53^WT^) and SY5Y-^sip53^ (SH-SY5Y). Both the parent and p53 knockdown cell line had the same pevonedistat sensitivity (Figure 1). There was also no difference in growth rates between p53^WT^ and knockdown cells (data not shown).

### 2.2. Pevonedistat-Induced Apoptosis Varies by p53 Status

**Figure 2:**
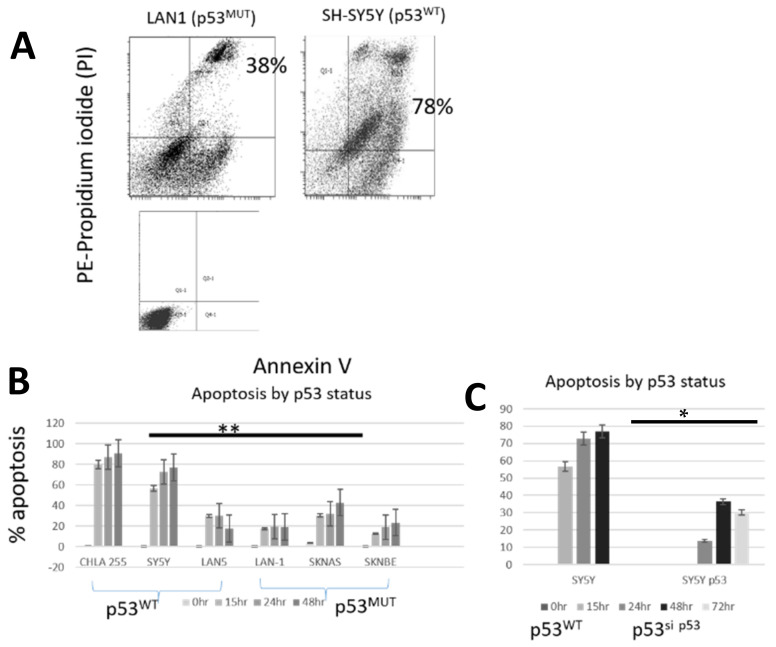
Apoptosis in neuroblastoma cell lines after treatment with pevonedistat at the IC50. (**A**) Annexin/PI staining in representative cell lines plus control. Annexin measured by +/+ i.e., positive for both annexin and PI. (**B**) Apoptosis by p53 status. (**C**) Apoptosis in SH-SY5Yand SH-SY5Y p53 knock down cell lines. ** *p* < 0.01; * *p* < 0.05. Data is representative of repeat experiments.

We used flow cytometry with Annexin V/PI to compare apoptosis after exposure to pevonedistat in three p53^WT^ and three p53^MUT^ neuroblastoma cell lines (example in Figure 2A). All six cell lines underwent apoptosis after treatment with pevonedistat (Figure 2B). However, the p53^MUT^ neuroblastoma cell line had fewer Annexin positive (mean +/− standard error) cells than the p53^WT^ cells (Figure 1B: p53^MUT^ median 19%, range 12–32% (right panel) vs. p53^WT^ median 73%, range 22–91% (left panel); *p* < 0.01) Apoptosis was also significantly decreased in the SH-SY5Y p53 knockdown cells compared to SH-SY5Y cells with p53^WT^ (median 30%, range 14–36% vs. 54%, range 58–77%; *p* < 0.05) (Figure 2C). Thus, neuroblastoma cells with intact p53 were more likely to undergo cell death through apoptosis. These data suggest that p53 status could influence mechanism of cell death.

### 2.3. Pevonedistat-Induced Cell Cycle Arrest Varies by p53 Status

Due to differences in apoptosis following treatment with pevonedistat, we hypothesized that p53 status might impact the mechanism of cell cycle arrest in neuroblastoma. Pevonedistat has been noted previously to have varying effects on cell cycle arrest depending on cell type, with different cell types arresting in G0/G1, G2-M or M-S depending on the cell type [1,2,10,11].

**Figure 3:**
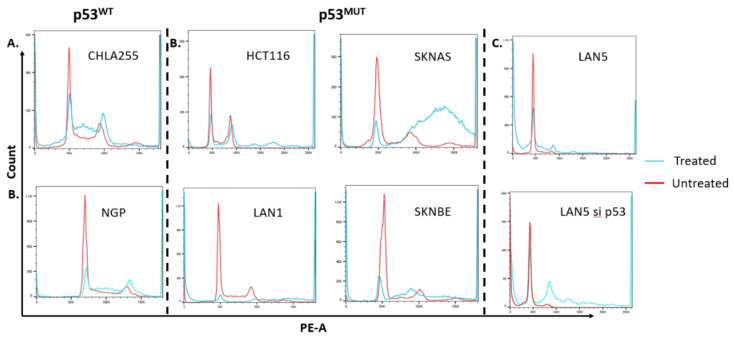
Cell cycle arrest as demonstrated by PI staining in neuroblastoma cell lines after treatment with pevonedistat. (**A**) Cell cycle arrest in p53 WT neuroblastoma cell lines that were untreated (red line) or after treatment with pevonedistat at Ic50 as noted in Figure 1 (blue line): G0/G1 arrest in p53^WT^ CHLA255 and NGP neuroblastoma cells. (**B**) Increased copy number accumulation in p53^MUT^ cell lines including colon cancer cell line HCT116 and the neuroblastoma cell lines SKNBE, LAN1 and SKNAS. (**C**) Cell cycle arrest in LAN5 and accumulation of high copy number cells in LAN5 p53 knockdown cell lines. Sub G1 fraction in LAN5 treated likely apoptosis as demonstrated in Figure 3B.

**Figure 4:**
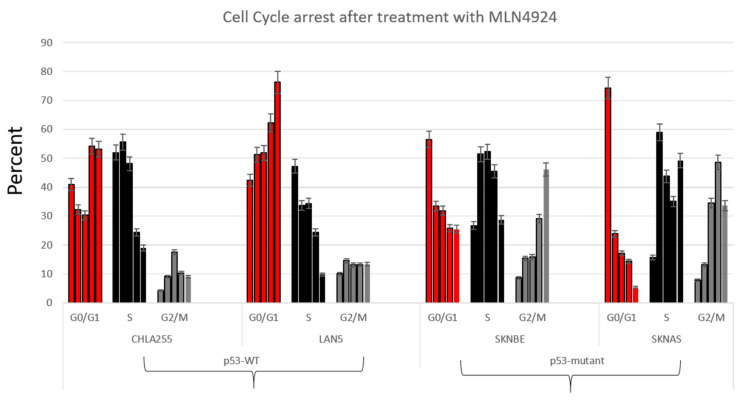
Cell cycle arrest as demonstrated by BrdU staining in neuroblastoma cell lines after treatment with pevonedistat. First bar: no treatment, second bar: 16 h MLN4924, third bar: 24 h MLN4924, fourth bar: 48 h MLN4924, fifth bar: 72 h MLN4924. Data is representative of repeat experiments.

To determine whether pevonedistat had different effects on ploidy or cell cycle arrest in p53^WT^ versus p53^MUT^ cells, we performed cell-cycle analysis using propidium iodide (PI) (Figure 3) and bromodeoxyuridine (BrdU) on neuroblastoma cell lines with different p53 status (Figure 4). There were low levels of sub-G1 signal in some cell lines following pevonedistat treatment. LAN5 had a more substantial sub-G1 signal that was not p53 dependent (Figure 3). To determine whether pevonedistat treatment resulted in cell cycle arrest or alteration of DNA content in neuroblastoma cells, we treated p53^MUT^ and p53^WT^ neuroblastoma cells with pevonedistat for 15 h, 24 h, 48 h and 72 h. Following pevonedistat treatment, the p53^MUT^ and p53^WT^ neuroblastoma cells expressed two distinct patterns of cell cycle arrest. The p53^WT^ cells (Figure 3, left panel) arrested in S phase and showed no evidence of increased DNA content (no peaks to the right of G2/M). The p53^MUT^ mutant cells SKNBE and SKNAS, in contrast (Figure 3, middle and right panels), showed no increase in S phase, and instead showed increases in G2/M along with increases in DNA content (peaks to the right of G2/M) consistent with rereplication.

To determine if differences in cell cycle arrest and the accumulation of increased DNA content prior to cell death were due to p53 status and not to other differences between neuroblastoma cell lines, we determined DNA content in two p53^WT^ neuroblastoma parent cells (LAN5) and compared DNA content to the same cells transduced with a p53 knockdown construct (Figure 3, right panel). These knockdowns also showed that cells with p53^WT^ arrested in S phase, whereas those with p53 knockdown developed cells with increased DNA content similar to the HCT116 control cell line. The selective development of high ploidy DNA suggests that the effects of pevonedistat on cell cycle arrest and the development of increased DNA content in vitro are p53 dependent.

**Figure 5:**
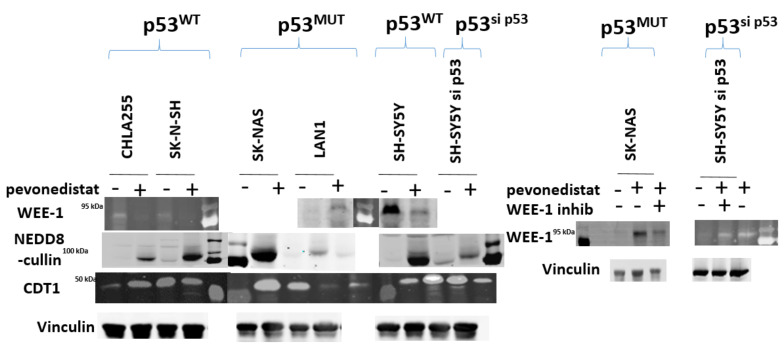
Immunoblot analysis of neuroblastoma cell lines after treatment with pevonedistat. p53^WT^ (cell lines CHLA255 and SKNSH), p53^MUT^ cell lines (SKNAS and LAN1), and p53 knockdown cell line (SH-SY5Y^si^-p53) were treated with pevonedistat at their IC50 for 48 h and protein expression was assessed for the proteins listed.

To further support the hypothesis that p53 resulted in different mechanisms of cell cycle arrest, we performed BrDU analysis on four neuroblastoma cell lines with varying p53 status (Figure 4). Similar to the different cell cycle patterns seen in Figure 2, neuroblastoma cells treated with pevonedistat followed by BrDU revealed two distinct patterns of cell arrest: p53^MUT^ cell lines arrested G2/M (Figure 4, right panel) whereas p53^WT^ cell lines arrested in G0/G1 (Figure 4, left panel). In p53^MUT^ cell lines SKNBE and SKNAS, the percentage of cells in G2/M increased from 8.2% to 46.1% and 7.8% to 48.6%, respectively, while the percentage of cells in G0/G1 and S decreased from over 70% to less than 25% following pevonedistat treatment. In contrast, the p53^WT^ cells (Figure 4, left panel) underwent G0/G1 arrest. In the p53^WT^ cell line LAN5, the percentage of cells in G0/G1 increased from 42% to 76%. In p53^WT^ cells, peak apoptosis occurred 24 h after pevonedistat treatment, whereas in p53^MUT^ cells, the peak effect occurred after 72 h of treatment. We performed immunoblots to assess the effects of pevonedistat on two CRL substrate proteins known to be involved in cell cycle progression, WEE1 and CDT1 (Figure 5). Since we observed differences in apoptosis and cell cycle arrest following pevonedistat treatment in neuroblastoma cells, we hypothesized that the effects of pevonedistat would differ for DNA replication proteins degraded by the CLN-ubiquitin system. Demonstrating target effect, all neuroblastoma cell lines treated with pevonedistat showed an increase in cullins due to the inhibition of their degradation (Figure 5, row 2). In p53^MUT^ cell lines, the cell cycle progression protein WEE1 increased with pevonedistat exposure. In p53^WT^ cell lines, however, the large amount of WEE1 at baseline decreased after 48 h of pevonedistat treatment. When p53^MUT^ or p53^si-p53^ cells were treated with both pevonedistat and a direct WEE kinase inhibitor (MK-1775), there was more accumulation of WEE1 after treatment with pevonedistat alone compared with the combination treatment (Figure 5).

CDT1, a cell cycle regulation protein, is also regulated by neddylation. Other studies have shown that treatment with pevonedistat inhibits CDT1 degradation, resulting in increased CDT1 concentration after treatment with pevonedistat. In this study, there was an increase in CDT1 in some neuroblastoma cell lines, but not in others, and the increase in CDT1 did not appear dependent on p53 status (Figure 5). This would suggest that the effects of p53 on pevonedistat are dependent on WEE1, but are not generalized to all CRL substrates involved in DNA regulation.

### 2.4. In Vivo Pevonedistat Results in Decreased Tumor Weight in Neuroblastoma Xenografts

**Figure 6:**
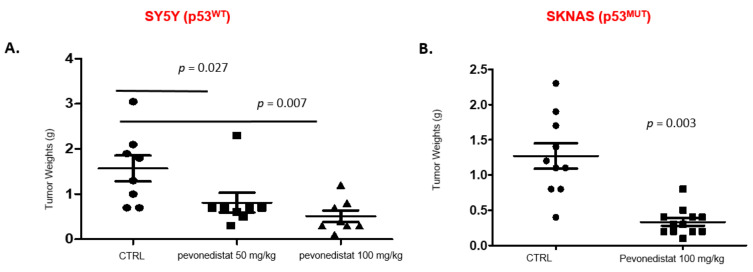
Tumor weights in two orthotopic xenograft models after treatment with pevonedistat. (**A**) Tumor weights in SY5Y orthotopic xenografts treated with either vehicle alone, 50 mg/kg of pevonedistat, or 100 mg/kg of pevonedistat. (**B**) Tumor weights in SKNAS orthotopic xenografts treated with either vehicle alone or 100 mg/kg of pevonedistat.

We then investigated the in vivo antitumor activity of pevonedistat. Using a well-established orthotopic neuroblastoma model, we generated xenograft tumors derived from p53 wild-type (SH-SY5Y) and p53 mutant (S-K-NAS) neuroblastoma cell lines (Figure 6). Luciferase-positive neuroblastoma cells were injected into the renal capsule of nude mice, and treatment was initiated two weeks after implantation. Vehicle control and pevonedistat (50 and 100 mg/kg) were given i.p. daily 6 days per week for 2 weeks and the effect on tumor growth was compared between groups at the end of treatment. SH-SY5Y (p53^WT^) tumor weight in the control group was 1.6 g + 0.8 g, vs. tumor weight of 0.8 g + 0.6 g and 0.5 g + 0.4 g in animals treated with 50 mg/kg or 100 mg/kg pevonedistat (*p* = 0.027 and *p* = 0.007, respectively) (Figure 6). S-K-NAS p53^MUT^ orthotopic xenografts also had decreased tumor weights when treated with 100 mg/kg of pevonedistat compared to the vehicle control (*p* = 0.003), indicating that pevonedistat is highly effective at decreasing tumor weight in preclinical models of neuroblastoma, independent of p53 status.

## 3. Discussion

These experiments are the first to demonstrate that neuroblastoma cells in vitro and in vivo in an orthotopic xenograft model with renal capsule injection are highly sensitive to the neddylation inhibitor pevonedistat. Our in vitro work also suggests that there are likely p53-dependent differences in the mechanism of neuroblastoma cell death. Despite p53-mediated differences in vitro, however, neuroblastoma cells in vivo appear to be highly sensitive to pevonedistat in orthotopic neuroblastoma models. Our in vitro data shows that wild type p53 neuroblastoma cells treated with pevonedistat underwent apoptosis, arrested in G0/G1, and expressed decreased WEE1, a regulator of the G2 checkpoint in response to DNA damage. In contrast, p53^MUT^ and p53 si-p53 neuroblastoma cells underwent limited apoptosis, developed increased DNA content consistent with rereplication, and had induction of WEE1 following pevonedistat treatment. Despite differences in mechanism, both p53^WT^ and p53^MUT^ responded to pevonedistat, with a three-fold decrease in tumor cell growth in orthotopic mouse models. This differs from other adult cancer types where p53 status correlated with sensitivity to MLN4924 and cells were more sensitive during S phase [1,10,11].

Neddylation affects a myriad of proteins, including p53, which are involved in cell cycle regulation and apoptosis. NEDD8 alters p53 activity through MDM2, resulting in the subsequent degradation of p53 via ubiquitination [12]. Several groups have shown that p53 activation and activity is dependent on neddylation status. For instance, NEDD8 alters p53-induced nucleolar signaling by altering the stability and localization of the ribosomal protein L11 [13] and recent work has shown that pevonedistat can activate p53 through the ribosomal-MDM2 pathway [14]. Though many proteins are neddylated, the specific microenvironment of each neuroblastoma cell line likely contributes to which proteins are impacted by neddylation inhibition. Microenvironment effects may also account for the relative absence of p53 status in pevonedistat in mouse models, where p53 status had little impact on pevonedistat sensitivity. Ongoing research is evaluating detailed neddylation and proteome-wide profiling may provide future insight into these effects [15,16].

Our data shows that pevonedistat has widely differing effects on neuroblastoma cells in vitro depending on p53 status. This finding is not unique to neuroblastoma, as differences in cell death induction has also been found in some adult malignancies. Several mechanisms of cell death action have been implicated in cells treated with pevonedistat [6,10,11,17,18]. Following cell cycle arrest, some cell types undergo apoptosis (e.g., colon carcinoma), whereas others undergo senescence. In Ewing sarcoma, pevonedistat’s effect on the cell cycle-dependent cell death is dependent on the accumulation of WEE1 [8]. In non-Hodgkin lymphoma (NHL), however, pevonedistat-induced cell death it is reliant on the accumulation of NF-κB [7].

Our data indicate that the mechanism of pevonedistat-induced cell death in neuroblastoma in vitro varies by p53 status. Those with p53^WT^ undergo G1/G0 arrest and subsequent apoptosis, whereas those with p53^MUT^ undergo S phase arrest, WEE1 accumulation, and rereplication. This is consistent with the literature that each cancer type exhibits a different mechanism of cell death after treatment with pevonedistat. However, this is the first indication that p53 status impacts the process of cell death in neuroblastoma following pevonedistat treatment. Pevonedistat was equally cytotoxic regardless of p53 status in neuroblastoma in vivo; however, differences in mechanism of action may affect more subtle interactions that could affect patient care. For instance, there may be differences in how pevonedistat reacts with other chemotherapy agents based on the p53 status of a patient’s tumor. Prior work has shown that patients with neuroblastomas with high NEDD8 gene expression have a significantly decreased relapse-free survival compared to patients without elevated NEDD8 expression [19]. Pevonedistat-induced NEDD8 inhibition would provide an exciting new mechanism to target neuroblastoma. Current clinical trials have shown that pevonedistat is well-tolerated with a favorable toxicity profile. The tolerability of pevonedistat in conjunction with our preclinical data suggest that pevonedistat warrants further investigation in patients with neuroblastoma.

## 4. Materials and Methods

### 4.1. Drugs

Pevonedistat was a kind gift from Millennium Pharmaceuticals, Inc. (now Takeda Pharmaceuticals, Cambridge, MA, USA). Pevonedistat was dissolved in DMSO to an initial concentration of 10 mM and stored at −80 °C. For each experiment, the drug was further diluted in phosphate-buffered saline (PBS) to reach the desired final concentration as shown in each figure. The WEE1 inhibitor MK-1775 (300 nm) (MedChemExpress, Monmouth Junction, NJ, USA) was added to cells in culture after 24 h of treatment with pevonedistat, and cells were cultured for an additional 24 h.

### 4.2. Cell Lines and Culture

The following neuroblastoma cell lines were used: CHP212, SH-SY5Y, S-K-NAS, S-K-NBE, LAN5, LAN1, CHLA255, and IMR-32. SH-SY5Y, S-K-NAS, S-K-NBE, S-K-N-SH, LAN5, and LAN1 were cultured in RPMI medium containing 10% bovine growth serum (BGS). CHLA255 were cultured in Iscove’s Modified Dulbecco’s Media (IMDM, Thermo Fisher, Waltham, MA, USA) with 20% BGS. CHP212 were cultured in Eagle’s Minimum Essential Medium with 10% BGS. For immunoblotting, apoptosis, and flow cytometry, cells were exposed to pevonedistat at their _IC50_ (Figure 1) for 16–72 h as noted. Commercial cell lines were obtained from ATCC and all cell lines underwent authentication using short tandem repeat (STR) DNA profiling to confirm their identity prior to use. All cell lines were confirmed mycoplasma negative at six-month intervals.

### 4.3. Lentiviral Packaging and Transduction

p53 knockdown cell lines were generated using a lentiviral pLSLP vector containing a short-hairpin RNA (shRNA) sequence targeting p53. Briefly, 293T cells were transfected with the pLSLPw construct along with packaging plasmids, pVSVG and pLV-CMV-delta 8.2, using lipofectamine 2000 (Invitrogen, cat#11668019, Waltham, Massachusetts, USA). Virus-containing supernatants were collected at 48 h and 72 h after transfection. Two p53 wild-type neuroblastoma cell lines, SY5Y and LAN5, were transfected with a lentiviral pLSLP vector containing a short-hairpin RNA (shRNA) sequence targeting p53. The neuroblastoma cells were transduced with lentivirus for 24 h with 8 mg/mL polybrene (cat# TR-1003, Sigma, St. Louis, MO USA). Seventy-two hours after transduction, cells were grown in media containing 1 µg/mL puromycin to select stably transduced cells containing the p53 knockdown plasmid. The result was formation of SY5Y and LAN5 cell lines which did not express p53 [20].

### 4.4. Cytotoxicity Assays

Neuroblastoma cells were seeded in duplicate on 96-multiwell plates and grown for 24 h prior to exposure to vehicle or increasing concentrations of pevonedistat (12–1000 nM). In some experiments, 300 nM of the Wee1 inhibitor MK-1775 was added 24 h after pevonedistat [21]. After 72 h of drug exposure (96 h if MK-1775 was added), 15 µL of [3-(4,5-dimethyl-thiazol-2yl)-2,5-diphenyl-tetrazolium bromide] (MTT) was added to each well. The plates were incubated with MTT at 37 °C for 4 h and medium was replaced with 150 µL of DMSO. Optical density was measured at 550 nm using an Anthos Analytical (Durham, NC, USA) microplate spectrophotometer and values were plotted on a log-linear curve. Survival was plotted using Kaleidagraph software and IC50s were determined using the Hill equation.

### 4.5. Immunoblots

After drug incubation, neuroblastoma cells were lysed in 50 mM Tris-Cl (pH 7.4), 150 mM NaCl, 1% NP40, 0.25% Na-deoxycholate, 1 mM PMSF, and 1 complete mini protease inhibitor cocktail (Roche). Immunoblot analysis was performed using the following antibodies: WEE1 (1:200, Santa Cruz Biotechnology, Dallas, TX, USA, sc5285), Cdt-1 (1:200, Cell Signaling Technology, Danvers, MA, USA, 3386S), vinculin (1:200, Abcam, Cambridge, MA, USA, EPR8185), and NEDD8 (1:200, Cell Signaling Technology, Danvers, MA, USA, 2745S, and a generous gift from Takeda Pharmaceuticals, Tokyo, Japan). Immunoblot analysis was performed as previously described [22].

### 4.6. Apoptosis Analysis

Apoptosis was determined using annexin V/PI staining by flow cytometry using an LSR2 (BD-LSR-II (SORP)). At least 20,000 cells were recorded for each time point. After incubation with pevonedistat (IC_50_) for 0–72 h, cells were harvested and resuspended in 500 µL of 1× Binding Buffer with 5 µL Annexin V-FITC and 5 µL of PI. Cells were incubated in the dark for 5 min at room temperature before analysis. Apoptosis was defined by having positivity to PI and Annexin V.

### 4.7. DNA Content and Cell Cycle Analysis

After pevonedistat incubation (0–72 h), cells were harvested and fixed with 70% ethanol overnight. Bromodeoxyruridine (BrdU) analysis was performed using a BrdU kit (BDBiosciences, San Jose, CA, USA) according to the manufacturer’s instructions. For cell cycle analysis, cells were trypsinized, collected, and resuspended by gentle vortex while drop wise adding 5 mL of 95% ethanol. Cells were fixed for 30 min and resuspended in 1 mL of diluted PI (final PI concentration 0.5 µg/mL). PI was reconstituted with PBS from a 500 µg/mL stock solution. RNase 1 mg/mL was added and cells were incubated at 37 °C for 30 min prior to flow cytometry analysis. Data were analyzed using FlowJo (FlowJo, LLC, Ashland, OR, USA).

### 4.8. In Vivo Experiments

All animal experiments were approved by the Baylor College of Medicine Institutional Animal Care and Use Committee (IUCAC). Orthotopic xenografts of human neuroblastoma cell lines (p53^WT^ and si-p53) were generated by injection under the renal capsule of an inoculum of 10^6^ SH-SY5Y cells in 0.1 mL of PBS. Two weeks post-implantation, mice were randomized in three treatment groups of ten mice each: vehicle control vs. pevonedistat at 50 mg/kg and 100 mg/kg {Smith, 2012 16/id; Soucy, 2009 35/id}. All drugs were given via intraperitoneal (i.p.) injection daily for 6 days per week for 2 weeks. Four weeks post-implantation, mice were sacrificed and tumors resected, weighed, and preserved in 4% paraformaldehyde overnight followed by dehydration with 25, 50, and 75% ethanol for immunohistochemistry analysis.

### 4.9. Statistics

Percent of apoptosis between cell lines was compared using the Mann–Whitney U test. For in vivo experiments, tumor weights were compared using Kruskal–Wallis analysis.

## Data Availability

Data is contained within the article.
